# The New Treatment for Consumption

**Published:** 1898-07

**Authors:** 


THE NEW TREATMENT FOR CONSUMPTION.—Dr. Cock's "Anti bacilli compound"—now called "anti-phimin"—(anti-tubercle). Readers of the TEXAS MEDICAL JOURNAL will doubtless recall an editorial in our April, 1897, issue, in which an account of this preparation was given, with the opinion that it is a rational remedy, and theoretically would seem to meet the indications in the earlier stages of consumption. They will doubtless be interested to hear something further from the remedy, which I said at the time, gave promise of good results.

Dr. Cock, who is a Texas physician—a graduate of Tulane—and in full fellowship with the Texas State Medical Association, and in excellent social and professional standing informs me that the old and strong New Orleans drug house, I. L. Lyons & Co., have undertaken the manufacture, under his formula—and sale of the remedy;—and that with their large capital and well known enterprise they will push it; having received such clinical reports upon it in the hands of prominent physicians as to satisfy them of its efficacy—not only in consumption, but in many pulmonary and stomach affections. It will be sold only *through the medical profession.* Dr. Cock has shown us reports of cases where remarkable results have followed the use of the remedy. By his permissson and that of Messrs. Lyons & Co. we publish one of these reports. It is from Dr. J. W. Daniel, of Houston. It will be found under the head of Publishers Notes, and attention is called to it.

